# International comparison of home dialysis uptake: a multi-registry analysis from the INTEGRATED Research Group

**DOI:** 10.1093/ckj/sfag271

**Published:** 2026-08-17

**Authors:** Annabel Boyer, Isabelle Ethier, David W Johnson, Karthik Tennankore, Thierry Lobbedez, Clémence Béchade, Cécile Couchoud, David Semple, Naoual El Ftouh, Oluwatoyin Idaomeh Ameh, Mark Lambie, Annie-Claire Nadeau-Fredette

**Affiliations:** Centre Universitaire des Maladies Rénales, CHU de Caen, Caen Cedex 9, France; U1086 INSERM—ANTICIPE— Centre Régional de Lutte Contre le Cancer, François Baclesse, Caen, France; Division of Nephrology, Department of Medicine, Centre Hospitalier de l’Université de Montréal, Montréal, QC, Canada; Health Innovation and Evaluation hub, Centre de Recherche du Centre Hospitalier de l’Université de Montréal, Montréal, QC, Canada; Australasian Kidney Trials Network, University of Queensland, Brisbane, Australia; Department of Kidney and Transplant Services, Princess Alexandra Hospital, Brisbane, Australia; Translational Research Institute, Brisbane, Australia; Division of Nephrology, Department of Medicine, Dalhousie University, Halifax, Nova Scotia, Canada; Nova Scotia Health, Halifax, Nova Scotia, Canada; Centre Universitaire des Maladies Rénales, CHU de Caen, Caen Cedex 9, France; U1086 INSERM—ANTICIPE— Centre Régional de Lutte Contre le Cancer, François Baclesse, Caen, France; UFR de Médecine, Normandie Université, Caen Cedex, France; Centre Universitaire des Maladies Rénales, CHU de Caen, Caen Cedex 9, France; U1086 INSERM—ANTICIPE— Centre Régional de Lutte Contre le Cancer, François Baclesse, Caen, France; UFR de Médecine, Normandie Université, Caen Cedex, France; Renal Epidemiology and Information Network (REIN) Registry, Biomedicine Agency, Saint-Denis-La-Plaine, France; Department of Renal Medicine, Auckland District Health Board, Auckland, New Zealand; Faculty of Medical and Health Sciences, University of Auckland, Auckland, New Zealand; Maisonneuve-Rosemont Hospital Research Center, Montreal, QC, Canada; Kidney Unit, Department of Renal Medicine, Royal Stoke University Hospital, Stoke-on-Trent, UK; Kidney Unit, Department of Renal Medicine, Royal Stoke University Hospital, Stoke-on-Trent, UK; School of Medicine, Keele University, Stoke-on-Trent, UK; Department of Medicine, Université de Montréal, Montreal, Canada; Division of Nephrology, Hôpital Maisonneuve-Rosemont, Montreal, Canada

**Keywords:** home dialysis, home haemodialysis, international variability, peritoneal dialysis

## Abstract

**Background:**

Despite advantages, home dialysis remains underutilized globally, with significant international variability. This study aims to analyse the pattern and uptake of home dialysis within the first year of dialysis across populations in different countries with similar access to kidney replacement therapy (KRT).

**Methods:**

This retrospective observational study utilized data from multiple national registries: ANZDATA (Australia–New Zealand), CORR (Canada), REIN (France), and UKRR (UK). We included adults over 18 starting dialysis between 1 January 2008 and 31 December 2018. The primary outcome was home dialysis initiation within the first year, visualized using cumulative incidences. Adjusted associations between patient characteristics and home dialysis initiation were analysed using clustered Cox regressions.

**Results:**

The study included 30 370 patients from ANZDATA, 50 682 from CORR, 103 962 from REIN, and 72 397 from UKRR. Home dialysis uptake at 12 months ranged from 41% (ANZDATA) to 13% (REIN). Transplant rates at 12 months varied from 1.1% (New Zealand) to 3.6% (UK), while 1-year mortality ranged from 5.5% (New Zealand) to 14% (France and Canada). Median age at dialysis initiation ranged from 59 years (New Zealand) to 71 years (France). Increased age was associated with a lower probability of home dialysis.

**Conclusions:**

This study highlights international differences in use of home dialysis within the first year of KRT. Patterns of conversion from facility haemodialysis to home dialysis were also different, underscoring the potential importance of healthcare systems and cultural factors within countries.

KEY LEARNING POINTS **What was known:**

- Home-based dialysis (PD and HHD) remains underused, with variability among high-income countries.
- Factors at patient, centre, and national levels, as well as competing events such as transplantation and death, may affect its adoption, but their relative impact remains unclear and warrants further study.

**This study adds**:

- We observed significant variations in the usage of home dialysis, with the cumulative incidence ranging from 41% (ANZDATA—Australia and New Zealand) and 32% (CORR—Canada) to 13% (REIN—France) and 26% (UKRR—UK).
- Older age and higher comorbidity burden was consistently associated with reduced home dialysis usage, with similar effect sizes across countries.
- We observed different patterns of transfer to home dialysis after the first months of starting KRT. Specifically, the conversion rate remained high for the first 3–4 months after KRT initiation in Australia, New Zealand and Canada, whereas it decreased immediately after the first month of dialysis in France and the UK.

**Potential impact**:

- The differing patterns of transition to home dialysis observed across countries highlight opportunities for improvement in healthcare policy and centre-level programmes, such as transitional care initiatives, to increase home dialysis uptake among patients initiating kidney replacement therapy with haemodialysis.

## INTRODUCTION

Despite its known advantages, home-based dialysis remains underutilized in many parts of the world. There is a wide international variability in home dialysis prevalence among high income countries, with up to 50% of patients on dialysis treated with home dialysis in New Zealand (NZ), around 25% in Australia and Canada, <20% in the UK and <10% in France [1]. For international initiatives such as the International Home Dialysis Consortium [2] or the EuroPD Policy Forum [3] to successfully address this variation, a better understanding of the drivers of this variability will be necessary.

The availability and use of home dialysis are associated with patient characteristics, such as age, sex, comorbidities, and socio-economic status [4–6], as well as with centre characteristics, such as number of incident dialysis patients, engagement with quality improvement projects, attitudes, and beliefs of the healthcare staff [4, 5, 7, 8]. Healthcare system factors, including such as kidney replacement therapy (KRT) availability, access, and affordability; cost and reimbursement differences between facility and home dialysis; regional cultural attitudes; policies favouring home dialysis; and provider financial incentives or penalties are all thought to impact home dialysis utilization at the national levels, although the relative impacts of these factors are not clear [3, 9–15]. Furthermore, variability in competing events, such as transplantation and transfer to facility-HD, may impact home dialysis initiation and prevalence.

The aim of this study was to analyse the uptake of home dialysis within the first year of dialysis, in each population of different countries with general availability of, and access to, KRT, testing the hypothesis that the uptake of home dialysis would differ. We specifically aimed to describe how variations in age, sex, and competing events may be associated with home dialysis uptake in different populations.

## MATERIALS AND METHODS

### Study population

This retrospective multi-national registry study included data from the Australia and NZ Dialysis and Transplant (ANZDATA) Registry, Canadian Organ Replacement Register (CORR), Réseau Épidémiologie et Information en Néphrologie (REIN—France), and United Kingdom Renal Registry (UKRR). All adults aged 18 years and older who initiated any dialysis modality for the first time in one of the participating countries between 1 January 2008 and 31 December 31 2018 (31 December 2017 for ANZDATA), were included. This timeframe has been deliberately selected to exclude the COVID-19 pandemic years (from 2019ṭo 2021), as this period introduced atypical practices in healthcare due to the global health crisis.

Characteristics of each registry are described in Supplementary Table S1. Patients receiving a pre-emptive transplant were excluded.

Patients were followed from the start of dialysis until the earliest occurrence of PD or HHD initiation, death or kidney transplantation. In the absence of occurrence of one of these events, follow up ended at 1 year after dialysis initiation.

### Events of interest

Our main event of interest was home dialysis initiation, defined as at least 1 day on home dialysis during the first year after dialysis initiation. Home dialysis initiation was characterized by the occurrence of either PD or HHD initiation (composite endpoint), PD initiation (HHD-censored), or HHD initiation (PD-censored). Death and kidney transplantation were considered as competing events.

For each patient, survival time was defined as the length of time between dialysis initiation and any of the events of interest, competing events or end of follow-up (12 months post dialysis initiation). For patients starting directly on home dialysis, this time was set to 1 day for the purpose of the analyses. Patients lost to follow-up were censored at the latest available date.

### Definition of variables

Start of home dialysis, and dates of death and/or kidney transplantation were retrieved from the registries.

Baseline characteristics were evaluated at the time of dialysis initiation and included age, sex, primary kidney disease, body mass index (BMI), comorbidities (including diabetes mellitus, chronic lung disease, coronary disease, peripheral vascular disease, cerebrovascular disease, and malignancy), and current smoking status. Baseline characteristics were captured according to standardized definitions used in each registry.

The main explanatory variable was the calendar time from date of dialysis initiation divided into 1-month intervals during the first year of dialysis, reflecting the patients’ trajectories divided by 1-month periods.

### Statistical analysis

Analyses were performed independently in each registry using identical definitions and statistical approaches. Each registry has the approval of their Ethics Committee. This study took place within the framework of these authorizations. This study was reported in accordance with the Strengthening the Reporting of Observational Studies in Epidemiology guidelines (STROBE 2008).

Categorical variables were described by their absolute numbers and percentages, while continuous variables were reported as medians and interquartile ranges (IQRs).

Home dialysis incidence was defined as the number of patients starting home dialysis (either PD or HHD) each year over the study period. Cumulative incidence functions were used to display the occurrence of the events over time.

Because the hazard of home dialysis initiation is not constant over time in the first year (high early hazard), we conducted clustered Cox analyses. This model assumes a constant hazard within short intervals [16]. In our case, the follow-up time was divided into monthly intervals, and the clustered Cox model was applied, treating each month as the unit of analysis and each patient as a cluster. Cause-specific hazard ratios (cs-HRs) and their 95% confidence intervals (CIs) were calculated using a clustered Cox regression model to explore the association between each variable and the events of interest [17]. All variables considered potentially relevant *a priori* and available in all registries were included in the multivariable models. Year of dialysis initiation was included in the multivariable analyses to assess the potential changes of the effect over the study period.

In a secondary analysis, to better describe the patterns in home dialysis uptake within the first year after facility-HD initiation (reflecting a switch to home dialysis), patients starting dialysis directly on home dialysis were excluded from the analyses. The percentage of patients transferring from facility-HD to either home dialysis (composite end-point), PD and HHD was then described by month after facility-HD initiation.

In most registries, there were ∼5%–10% missing data on the main variables of interest, which were assumed to be missing at random, thereby enabling the performance of a complete case analysis [18]. A primary analysis was performed including all variables available in all registries, independently of the proportion of missing data. Because of a high proportion (over 50%) of missing data on some comorbidities, notably the BMI in the UKRR, a sensitivity analysis including only variables with ∼10% missing data was performed to limit the proportion of patients excluded from the analyses.

Statistical analyses were performed with R version 4.0.2 (R Foundation for Statistical Computing, Vienna, Austria) for REIN, SAS software version 9.4 (SAS Institute, Inc., Cary, NC, USA) for CORR and Stata/SE software (version 15.1, StataCorp, College Station, TX, USA) for ANZDATA and UKRR.

## RESULTS

### Patient characteristics

**Figure 1:**
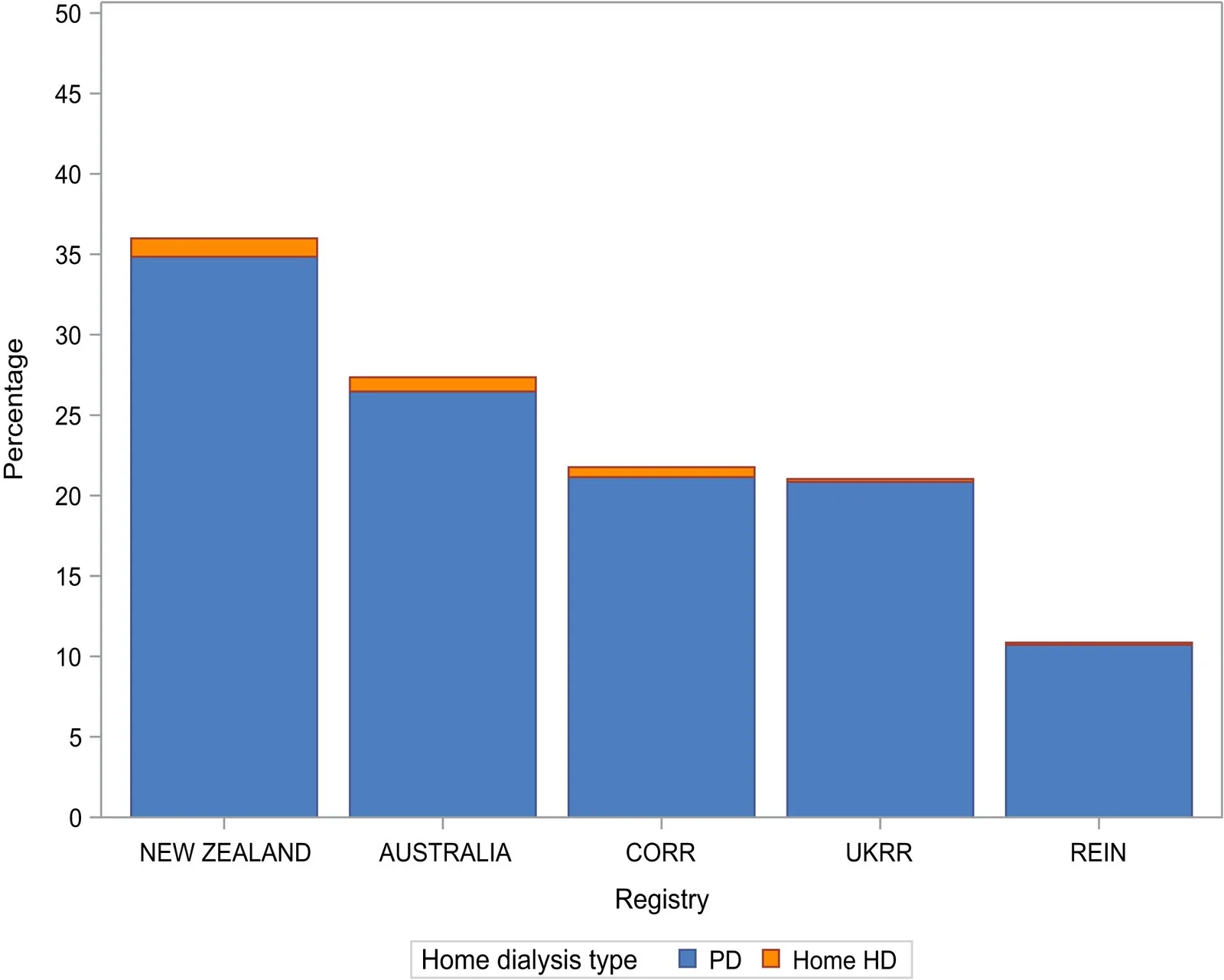
Percentage of patients directly initiated on home dialysis, stratified by PD and HHD, by registry among all dialysis initiation. PD, peritoneal dialysis, HHD, home haemodialysis.

In the ANZDATA registry, 30 370 patients were included (25 199 in Australia and 5171 in NZ), 50 682 in the CORR, 103 962 in the REIN, and 72 397 in the UKRR. Of these, 35% were on home dialysis at cohort entry in the ANZDATA-Australia, 36% in the ANZDATA-NZ, 22% in the CORR, 11% in the REIN, and 21% in the UKRR. The proportion of patients that directly initiated on home dialysis, stratified by PD and HHD and by registry, is represented in Fig. 1.

Patient characteristics at cohort entry by registry are provided in Table 1. Characteristics had some similarities across the different registries, with most individuals being men aged 65 or older. The lowest median age was observed in the ANZDATA-NZ cohort, while the highest median age was observed in the REIN cohort. Table S2 provides patients’ characteristics according to the type of first home dialysis (e.g. PD or HHD), by registry. Characteristics of incident patients on home dialysis and those who transferred to home dialysis, by registry, are provided in Table S3.

**Table 1:** Patient characteristics by registry at KRT start.

Variable	ANZDATA-Australia (*n* = 25 199)	ANZDATA-NZ (*n* = 5171)	CORR (*n* = 50 682)	REIN (*n* = 103 962)	UKRR (*n* = 72 397)
First dialysis modality					
In centre haemodialysis	16 445 (65)	3310 (64)	39 651 (78)	92 674 (89)	57 175 (79)
PD	8472 (34)	1802 (35)	10 716 (21)	11 267 (11)	15 099 (21)
HHD	282 (1)	59 (1)	315 (1)	21 (0.02)	123 (0.2)
Age at dialysis initiation, *median (IQR)*	63 (51–73)	59 (49–68)	67 (57–77)	71 (60–80)	66 (53–75)
Sex, male	15 627 (62)	3128 (60)	31 255 (62)	66 361 (64)	45 707 (63)
Primary kidney disease					
Diabetes	9451 (37)	2639 (51)	19 135 (38)	23 141 (22)	18 877 (26)
Glomerulonephritis	4988 (20)	1019 (20)	6304 (12)	11 306 (11)	8838 (12)
Vascular or hypertensive nephropathy	3624 (14)^a^	514 (10)^a^	8101 (16)	28 251 (27)	9329 (13)
Other	6679 (27)	974 (19)	13 659 (27)	41 264 (40)	32 731 (45)
Missing	457 (2)	25 (0.48)	3483 (7)	0 (0)	2622 (4)
Comorbidities					
Diabetes	12 536 (50)	3001 (58)	26 932 (53)	44 308 (43)	18 877 (26)
Missing	162 (1)	9 (0.2)	1860 (4)	785 (8)	31 192 (43)
Pulmonary disease	3179 (13)	762 (15)	6009 (12)	15 639 (15)	3178 (4)
Missing	196 (1)	8 (0.2)	3446 (7)	3121 (3)	28 973 (40)
Coronary disease	8392 (33)	1383 (27)	9256 (18)	26 492 (25)	8461 (12)
Missing	195 (1)	9 (0.2)	3104 (6)	2933 (3)	32 131 (44)
Peripheral vascular disease	4471 (18)	701 (14)	7698 (15)	20 350 (20)	5143 (7)
Missing	199 (1)	8 (0.2)	3275 (7)	3788 (4)	29 557 (41)
Cerebrovascular disease	2787 (11)	548 (11)	6473 (13)	11 345 (11)	13 875 (19)
Missing	193 (1)	9 (0.2)	3034 (6)	2 563 (2)	32 977 (46)
Malignancy	2803 (11)^b^	462 (9)^b^	7210 (14)	11 681 (11)	5730 (8)
Missing	0 (0)	0 (0)	4146 (8)	2475 (2)	31 725 (44)
Current smoker	3050 (12)	790 (15)	7244 (14)	36 445 (35)	5070 (7)
Missing	553 (2)	27 (1)	4453 (9)	16 712 (16)	33 467 (46)
BMI					
<20 Kg/m²	680 (3)	67 (1)	3768 (8)	8166 (8)	52 (0.1)
20–24.9 Kg/m²	7407 (29)	1054 (20)	12 799 (25)	29 030 (28)	200 (0.3)
25–29.9 Kg/m²	7809 (31)	1504 (29)	13 839 (27)	26 385 (25)	251 (0.3)
30 Kg/m² and over	8611 (34)	2472 (48)	15 211 (30)	19 754 (19)	271 (0.4)
Missing	692 (3)	74 (2)	5065 (10)	20 627 (20)	71 623 (99)
Year of dialysis initiation					
2008–2011	9307 (37)	1971 (38)	16 399 (32)	33 843 (32)	24 799 (34)
2012–2015	10 251 (41)	2081 (40)	18 746 (37)	39 058 (38)	26 212 (36)
2016–2018	5641 (22)^c^	1119 (22)^c^	15 537 (31)	31 061 (30)	21 386 (30)

KRT, kidney replacement therapy; NZ, New-Zealand; CORR, Canadian Organ Replacement Register; REIN, Réseau Epidémiologique et Information en Néphrologie; UKRR, United Kingdom Renal Registry; PD, peritoneal dialysis; HHD, home haemodialysis; and BMI, body mass index.

a: hypertensive nephropathy; b: excluding skin cancer; and c: 2016–2017.

Within the first year of dialysis initiation in the ANZDATA-Australia, 10 245 (41%) patients initiated home dialysis, while 1841 (7%) patients died and 636 (3%) patients received kidney transplantation. Those numbers were 3122 (60%), 278 (5%), and 55 (1%), respectively, in the ANZDATA-NZ; 16 075 (32%), 7137 (14%), and 628 (1%) in the CORR; 13 730 (13%), 15 297 (15%), and 3448 (3%) in the REIN; and 19 150 (27%), 8356 (12%), and 2612 (4%) in the UKRR.

### Home dialysis initiation within the first year after dialysis initiation

**Figure 2:**
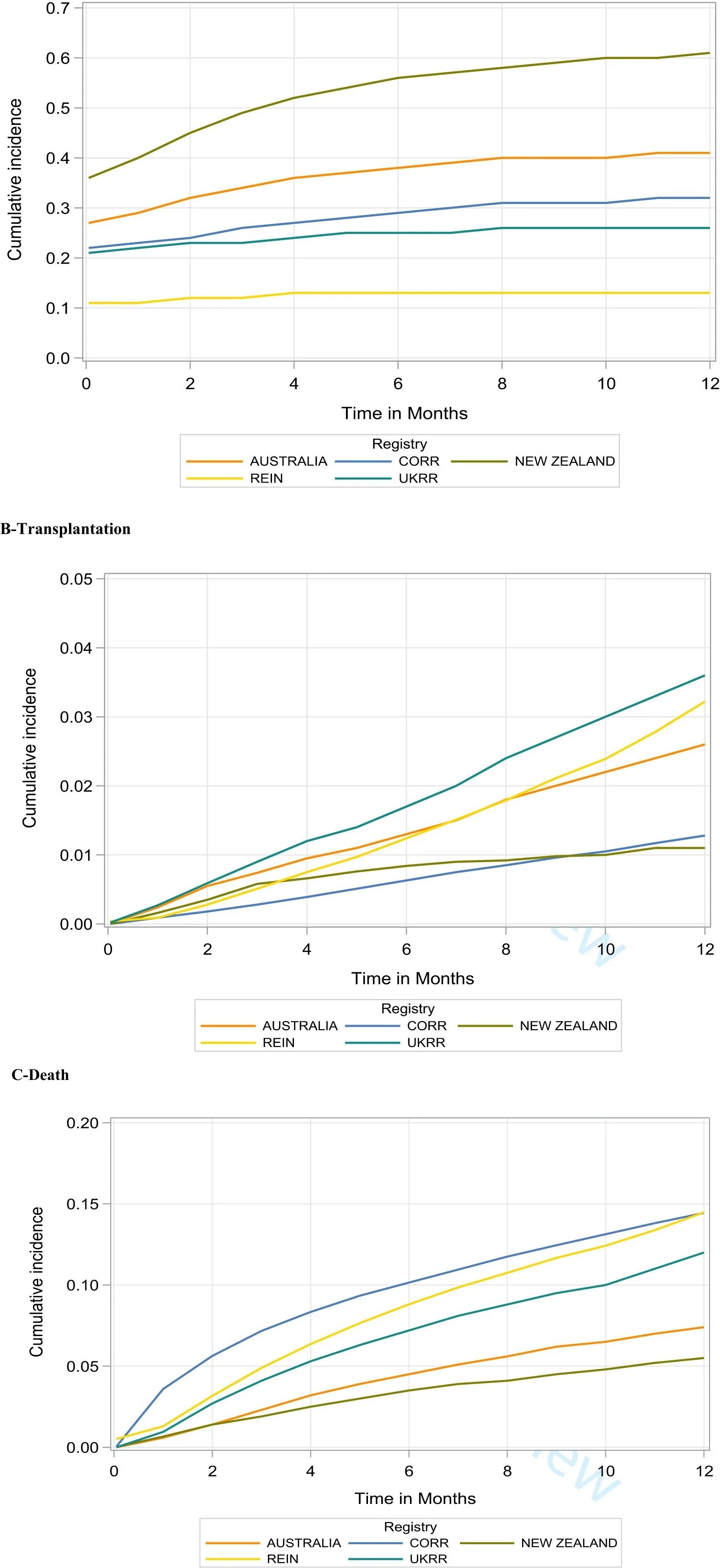
Cumulative incidence curves of the probability of starting home dialysis (a), transplantation (b), and death (c), for the different registries. CORR, Canadian Organ Replacement Registry; REIN, Reseau Epidémiologique et Informatique en Néphrologie; UKRR, United Kingdom Renal Registry.

**Figure 3:**
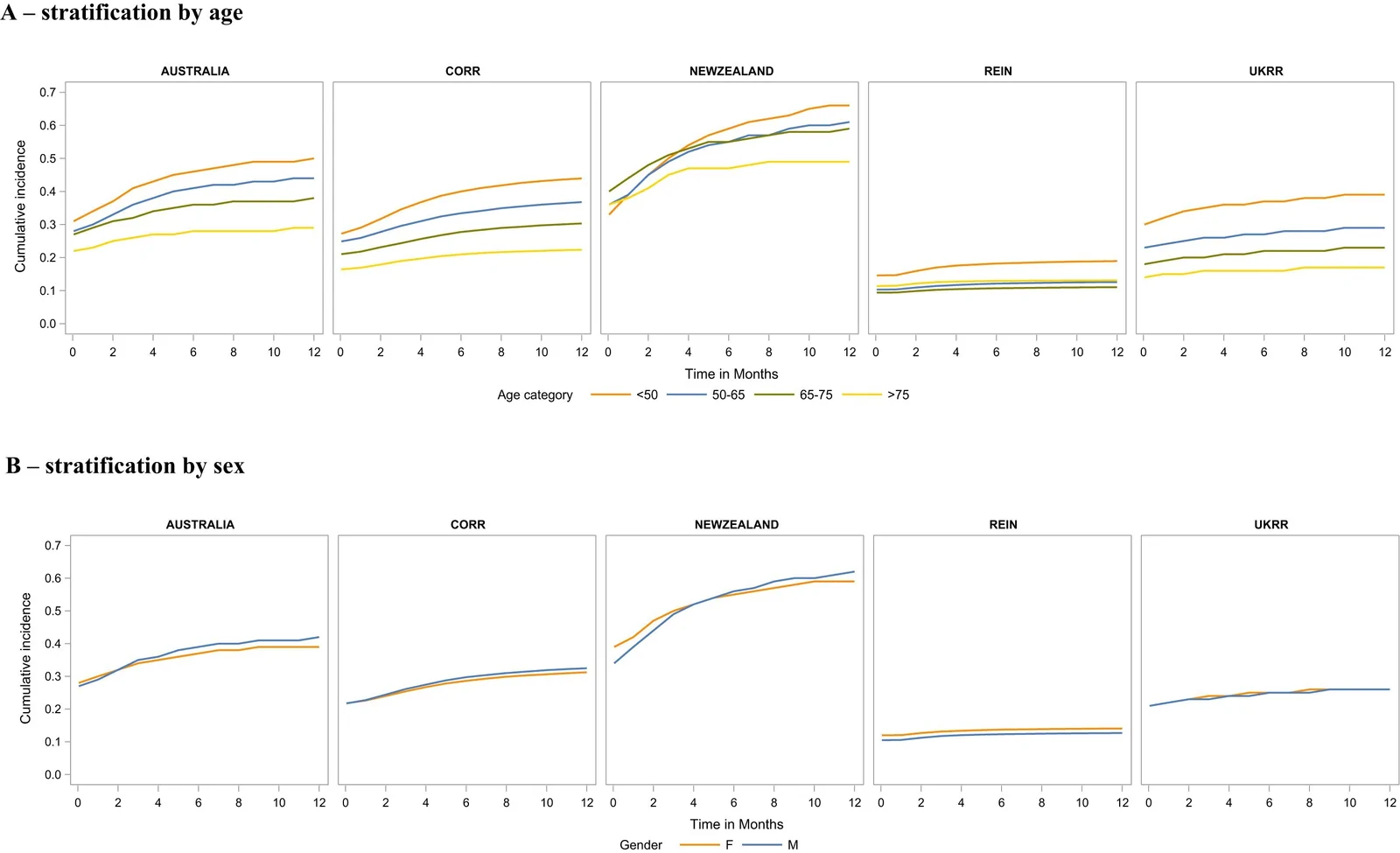
Cumulative incidence curves of the probability of starting home dialysis stratified by age (a) and sex (b) for the different registries. CORR, Canadian Organ Replacement Registry; REIN, Reseau Epidémiologique et Informatique en Néphrologie; UKRR, United Kingdom Renal Registry; F, female; and M, Male.

Home dialysis cumulative incidences at 12 months after dialysis initiation were 41%, 61%, 32%, 13%, and 26% in the ANZDATA-Australia, ANZDATA-NZ, CORR, REIN, and UKRR, respectively. Specific to modality, incident HHD use at 1 year ranged from 0.26% in France to 10% in NZ. Incident PD use at 1 year ranged from 13% in France to 50% in NZ. Cumulative incidence curves of the probability of starting home dialysis within the first year after dialysis initiation, for each registry, are displayed in Fig. 2a, and stratified by age and by sex, in Fig. 3a and b, respectively.

**Figure 4:**
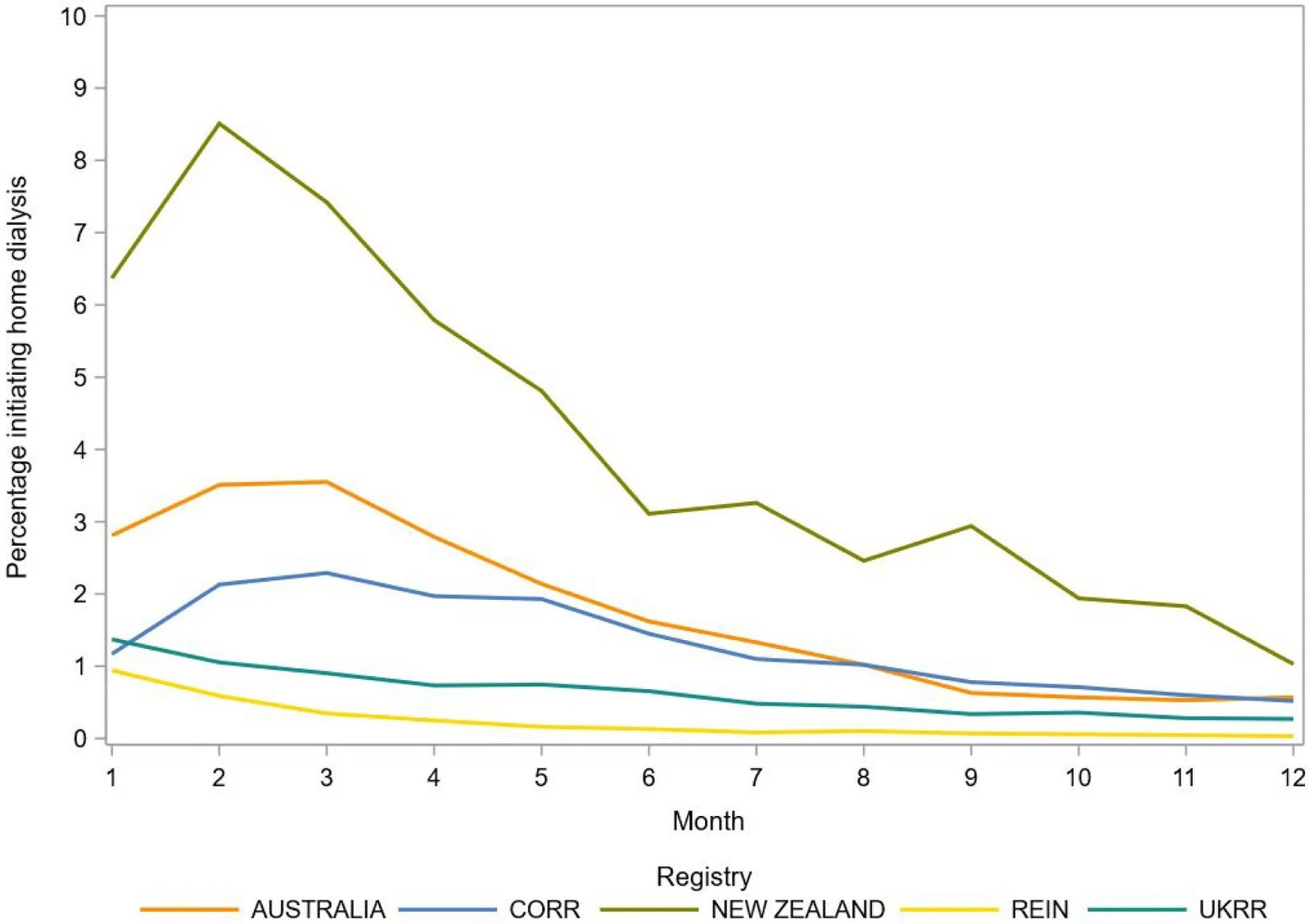
Percentage of patients transferred from facility-based dialysis to home dialysis by month, excluding patients starting on home dialysis, within the first year of haemodialysis initiation. Results are expressed in percentages, censored for death/transplantation/loss to follow-up/kidney recovery CORR, Canadian Organ Replacement Registry; REIN, Reseau Epidémiologique et Informatique en Néphrologie; and UKRR, United Kingdom Renal Registry.

Most patients initiated home dialysis during their first month of dialysis in all countries. In the ANZDATA (Australia and NZ) and the CORR, conversion to home dialysis remained high for the first 3–4 months after dialysis start. In contrast, in the REIN and the UKRR, home dialysis initiation decreased immediately after the first month of dialysis. Proportions of patients transferring from facility-HD to home dialysis, by month and by registry, excluding patients starting directly on home dialysis, are represented in Fig. 4.

In the adjusted clustered Cox regression, increasing age was associated with a decreasing relative hazard of starting home dialysis within the first year of dialysis across all registries, with the greatest impact observed in the UK. In France, the pattern differed slightly, with the >75 age group having a higher estimated cs-HR for home dialysis (cs-HR 0.70; 95% CI 0.66–0.75) compared to the 65–75 age group (cs-HR 0.62; 95% CI 0.58–0.67). Female sex was associated with a higher probability of home dialysis initiation in the REIN registry, while the opposite was observed in the CORR and the ANZDATA-Australia registries (Table 2). Comorbid conditions had similar effect estimates between countries, as did primary kidney disease categories when comorbid conditions were not included as covariates (Table S4). Initiating dialysis in a more recent era was associated with a higher probability of home dialysis initiation in all registries except REIN and ANZDATA-NZ (Table 2). Sensitivity analyses, which excluded variables with significant missing data, yielded similar results (Table S4).

**Table 2:** Adjusted clustered Cox model of home dialysis initiation within 1 year after dialysis initiation.

	ANZDATA-Australia	ANZDATA-NZ	CORR	REIN	UKRR
Explanatory variable	HR (95% CI)	HR (95% CI)	HR (95% CI)	HR (95% CI)	HR (95% CI)
Interval in month between dialysis initiation and home dialysis initiation					
Month 1	12.0 (11.0–13.0)	7.95 (6.98–9.05)	11.8 (10.9–12.8)	15.9 (14.4–17.5)	27.7 (25.5–30.2)
Month 2	Reference	Reference	Reference	Reference	Reference
Month 3	0.96 (0.86–1.08)	0.79 (0.65–0.95)	1.04 (0.93–1.17)	0.84 (0.74–0.97)	0.83 (0.73–0.93)
Month 4	0.73 (0.64–0.82)	0.56 (0.46–0.69)	0.92 (0.82–1.03)	0.44 (0.37–0.53)	0.65 (0.57–0.74)
Month 5	0.53 (0.46–0.61)	0.44 (0.35–0.54)	0.90 (0.80–1.02)	0.38 (0.31–0.45)	0.65 (0.57–0.74)
Month 6	0.39 (0.33–0.45)	0.26 (0.20–0.35)	0.69 (0.60–0.78)	0.19 (0.14–0.24)	0.56 (0.48–0.63)
Month 7	0.30 (0.25–0.36)	0.26 (0.20–0.35)	0.52 (0.45–0.60)	0.16 (0.12–0.20)	0.39 (0.34–0.46)
Month 8	0.23 (0.19–0.28)	0.19 (0.14–0.26)	0.44 (0.37–0.51)	0.17 (0.13–0.22)	0.35 (0.30–0.42)
Month 9	0.14 (0.11–0.17)	0.22 (0.17–0.30)	0.36 (0.31–0.43)	0.11 (0.08–0.16)	0.27 (0.23–0.32)
Month 10	0.12 (0.09–0.15)	0.14 (0.10–0.20)	0.33 (0.28–0.40)	0.10 (0.07–0.14)	0.28 (0.23–0.33)
Month 11	0.11 (0.09–0.14)	0.13 (0.09–0.18)	0.28 (0.23–0.34)	0.07 (0.05–0.11)	0.22 (0.18–0.26)
Month 12	0.12 (0.09–0.15)	0.07 (0.04–0.11)	0.14 (0.10–0.21)	0.10 (0.07–0.15)	0.20 (0.17–0.25)
Age at dialysis initiation					
<50	Reference	Reference	Reference	Reference	Reference
50–65	0.95 (0.91–0.99)	1.01 (0.93–1.09)	0.93 (0.89–0.97)	0.70 (0.66–0.75)	0.76 (0.74–0.79)
66–75	0.82 (0.77–0.86)	0.96 (0.88–1.05)	0.79 (0.75–0.83)	0.62 (0.58–0.67)	0.60 (0.58–0.62)
>75	0.58 (0.55–0.62)	0.71 (0.61–0.83)	0.56 (0.53–0.59)	0.70 (0.66–0.74)	0.44 (0.42–0.45)
Female sex	0.92 (0.88–0.95)	1.01 (0.94–1.07)	0.96 (0.93–0.99)	1.17 (1.11–1.22)	1.00 (0.97–1.03)
Primary kidney disease					
Diabetes	Reference	Reference	Reference	Reference	Reference
Glomerulonephritis	1.13 (1.06–1.21)	0.95 (0.82–1.11)	0.98 (0.91–1.04)	1.36 (1.25–1.48)	1.44 (1.38–1.50)
Vascular or hypertensive nephropathy	1.08 (1.00–1.16)^a^	0.99 (0.84–1.17)^a^	0.93 (0.87–1.00)	1.06 (0.98–1.14)	1.21 (1.15–1.27)
Other	0.96 (0.89–1.03)	0.87 (0.74–1.01)	0.71 (0.66–0.75)	1.07 (0.99–1.16)	1.12 (1.09–1.17)
BMI					
<20 Kg/m²	0.91 (0.84–0.99)	0.86 (0.72–1.02)	0.91 (0.85–0.97)	0.76 (0.70–0.82)	0.87 (0.53–1.41)
20–24.9Kg/m²	Reference	Reference	Reference	Reference	Reference
25–29.9 Kg/m²	1.02 (0.98–1.07)	0.98 (0.90–1.07)	1.04 (1.00–01.08)	1.09 (1.04–1.14)	1.06 (0.79–1.41)
30 Kg/m² and over	0.83 (0.79–0.87)	0.73 (0.67–0.79)	0.88 (0.84–0.92)	0.78 (0.74–0.83)	0.70 (0.51–0.96)
Comorbidities					
Diabetes	0.85 (0.80–0.90)	0.74 (0.65–0.86)	0.79 (0.74–0.84)	0.84 (0.79–0.89)	0.79 (0.73–0.84)
Pulmonary disease	0.77 (0.73–0.82)	0.82 (0.75–0.91)	0.67 (0.62–0.71)	0.79 (0.74–0.85)	0.77 (0.71–0.84)
Coronary disease	0.87 (0.83–0.91)	0.88 (0.81–0.95)	0.85 (0.81–0.89)	1.11 (1.05–1.17)	1.04 (0.97–1.11)
Peripheral vascular disease	0.86 (0.81–0.91)	0.95 (0.86–1.06)	0.79 (0.74–0.83)	0.96 (0.90–1.02)	0.92 (0.85–0.98)
Cerebrovascular disease	0.92 (0.87–0.99)	0.92 (0.82–1.03)	0.80 (0.75–0.85)	1.01 (0.94–1.08)	1.00 (0.94–1.07)
Malignancy	0.74 (0.69–0.79)^b^	0.90 (0.80–1.01)^b^	0.79 (0.75–0.83)	0.62 (0.57–0.67)	0.88 (0.83–0.93)
Current smoker	0.82 (0.78–0.87)	0.89 (0.82–0.98)	0.82 (0.78–0.86)	1.05 (0.99–1.10)	0.99 (0.94–1.05)
Year of dialysis initiation					
2008–2011	Reference	Reference	Reference	Reference	Reference
2012–2015	1.11 (1.06–1.15)	1.03 (0.96–1.11)	1.12 (1.07–1.16)	1.02 (0.97–1.07)	1.03 (1.01–1.07)
2016–2018	0.99 (0.94–1.04)^c^	1.01 (0.93–1.10)^c^	1.24 (1.19–1.30)	0.95 (0.90–1.01)	1.05 (1.02 -1.09)

NZ, New Zealand; CORR, Canadian Organ Replacement Register; REIN, Réseau Epidémiologique et Information en Néphrologie; UKRR, United Kingdom Renal Registry; BMI, body mass index.

a: hypertensive nephropathy; b: excluding skin cancer; and c: 2016–2017.

### Specific initiation of PD or HHD within the first year after dialysis initiation

When the analysis was restricted to PD initiation, the results remained consistent with the primary analysis. Female sex was associated with a higher probability of PD initiation in the ANZDATA-NZ and the REIN but the association was not significant in the other registries (Tables S5 and S6).

Similarly, predictors of HHD initiation within the first year of dialysis were consistent with overall home dialysis initiation, with the exception of female sex which was associated with a lower probability of HHD initiation in all registries except the REIN and the UKRR. The likelihood of starting HHD decreased more significantly with advancing age. The effect size of associations between comorbidities and HHD initiation was heightened. Starting dialysis in a more contemporary era was associated with a higher probability of HHD initiation in the CORR, UKKR, and especially REIN, while the opposite was observed in the ANZDATA (NZ and Australia). HHD initiation was more likely to happen within the first 3–6 months after dialysis initiation in all registries (Tables S7 and S8).

Figure S1a and b represents the proportion of patients who transferred from facility-based dialysis to PD and HHD respectively, when excluding patients starting directly on home dialysis from the analysis, over time, for each registry.

### Competing events: transplantation and death within the first year after dialysis initiation

At 1 year after dialysis initiation, the cumulative incidences of transplantation were 2.6% in the ANZDATA-Australia, 1.1% in the ANZDATA-NZ, 1.3% in the CORR, 3.2% in the REIN, and 3.6% in the UKRR. The cumulative incidences of death at 1 year after dialysis initiation were 7.4% in the ANZDATA-Australia, 5.5% in the ANZDATA-NZ, 14% in the CORR and REIN, and 12% in the UKRR. Cumulative incidence curves of the probabilities of transplantation and death within the first year after dialysis initiation, for each registry, are displayed in Fig. 2b and c, respectively, and the combined incidence is displayed in Table 3.

**Table 3:** Competing events and home dialysis uptake by country.

Country	Home dialysis cumulative incidence (%) by 12 months after KRT start	Cumulative incidence (%) of death or kidney transplantation by 12 months after KRT start
**New Zealand**	61	6.6
**Australia**	41	10.0
**Canada**	32	15.3
**UK**	26	15.6
**France**	13	17.2

KRT, kidney replacement therapy and UK, United Kingdom.

## DISCUSSION

This multi-registry study provides valuable insights into the international variability and patterns of home dialysis uptake within the first year of dialysis, highlighting significant disparities across several high-income countries with broadly similar reimbursement strategies. The incidence of home dialysis within the first year was the highest in ANZDATA-NZ (61%) and the lowest in REIN (13%), and conversion to home dialysis was high for the first 3–4 months after dialysis start in ANZDATA and CORR while it decreased immediately after dialysis start in the UKKR and REIN. Patient characteristics and competing events varied between countries in our analyses; however, their contribution to the differences observed in the initiation of (or conversion to) home dialysis over the first year of dialysis remains unclear. These findings underscore the need for a deeper understanding of the factors influencing home dialysis adoption, including the importance of healthcare policy, care delivery models, and cultural context.

Our analysis of the cumulative incidence of home dialysis align closely with existing literature based on the prevalence of home dialysis, with NZ leading at around 50% of prevalent dialysis patients using home dialysis, followed by Australia and Canada at around 25%, and the UK and France below 20% and 10%, respectively [1, 19–22].

The potential impact of competing events for home dialysis patients was highlighted in an international comparison where, relative to the USA, PD patients in the UK were 4 times more likely to receive a kidney transplant and patients in Japan were 5 times less likely [23]. This suggests that competing event rates may significantly affect prevalence rates. To address this, we analysed home dialysis incidence (rather than prevalence) and adjusted for competing events. Death and transplant rates varied by country, with the biggest difference between France and NZ: a 10.6% difference in competing events versus a 48% difference in home dialysis incidence. Pre-emptive transplants, excluded from the analyses due to inconsistent reporting, represented 2.7% (Australia) to 6.6% (UK) of incident KRT cases in 2022 [19–22]. The proportion of competitive events differed between countries, but this difference was much smaller than the difference observed in the use of home dialysis. Thus, the variation in competitive events alone appears to only partially account for the disparity in home dialysis utilization.

Differences in home dialysis uptake may also reflect variations in the KRT incident population. While older age is consistently linked to lower home dialysis use [5, 6], a finding replicated here, Australia and NZ showed the youngest KRT populations and the highest home dialysis usage. This younger KRT population in NZ has been previously reported, as Māori patients are known to develop kidney disease at a younger age and initiate KRT earlier compared to non-Māori patients [24]. However, there were large differences in home dialysis still apparent in age-stratified groups. Patient preferences and availability of comprehensive pathways for conservative treatments, which vary significantly between countries [25–27], could also influence home dialysis adoption.

A higher comorbidity burden is also consistently associated with reduced home dialysis usage [5, 6, 11]. This finding was replicated in this study, with similar effect estimates across all registries, but the KRT population in the countries with the highest home dialysis usage had similar or higher levels of comorbidity.

Sex based differences in access to home dialysis have been described previously [28, 29], but it has not been clear whether there is any consistent difference. This inconsistency was also evident in this analysis, with female sex associated with worse, equal and better access to home dialysis depending on the country. The largest effect estimate was for France, where females showed greater home dialysis usage.

When describing home dialysis usage, other factors beyond patient characteristics need to be considered [3, 11]. In France, uniquely among the studied countries, physician reimbursement is tied to dialysis modality, with private-sector nephrologists (covering ∼50% of patients) incentivized away from home dialysis. France also has healthcare provider reimbursement that favours facility based HD [30]. This suggests that the low rate of home dialysis usage in France relative to the other countries may be mostly due to differences in reimbursement. The other countries within the study do not have financial disincentives for home dialysis and have a high proportion of dialysis patients on home dialysis [1], suggesting that financial disincentives via physician reimbursement may hinder broader home dialysis adoption.

In countries without financial disincentives, large differences in home dialysis usage remained, suggesting that other issues had a large impact. Policies may contribute to these variations. France, e.g. supports assisted PD with registered nurses [31], potentially explaining why the older KRT patients in France had a relatively small reduction in home dialysis usage. However, most included countries lacked formal policies such as ‘PD first’, suggesting cultural factors may play a role. This aligns with findings in England, where cultural factors have been shown to drive substantial variability in home dialysis uptake between centres [8]. Centres where staff demonstrate a consistent belief in the benefits of home dialysis and practised ‘presumed eligibility’ had higher levels of home dialysis usage, underpinned by greater engagement in quality improvement [8]. While the Inter-CEPt study identified some of the issues contributing to cultural differences between centres within a country, these may or may not contribute to differences between countries, and there may well be other differences as well.

Healthcare organization may also differ between countries. While this study did not include data on late referrals, which has been associated with a decreased probability of home dialysis [32, 33], unplanned dialysis initiations average around 30% across registries [19–21], except in Australia (15%) and NZ (13%) [22]. Interestingly, we observed different patterns of transfer to home dialysis after the first months of starting KRT. Specifically, the conversion rate remained high for the first 3–4 months after KRT initiation in Australia, NZ, and Canada, whereas it decreased immediately after the first month of dialysis in France and the UK. Healthcare policies and nephrology service structures, such as pre-dialysis care programmes [34], frequent reassessment of patients initiating facility-HD, and transitional care units [35–37] (or community house haemodialysis [38–40] in NZ), are likely to influence home dialysis usage. These structures, more common in Canada, Australia, and NZ, may partly explain differences in early conversion from facility-HD to home dialysis. Implementation of such programmes and units should be further promoted worldwide, in order to increase transfer to home dialysis after KRT initiation, notably in the context of unplanned dialysis initiation.

While our study offers a comprehensive overview of home dialysis uptake across multiple registries, it has some limitations. The lack of standardized definitions may have introduced variability in the results, and there was notable uncertainty surrounding the definition of HHD training, which may have contributed to variations in how training was recorded. Data on pre-emptive transplant, an important competing risk to home dialysis, were unavailable in several registries. Due to data governance issues, we were unable to combine the different registry data in one analytical database. While the independent analysis of each dataset at the respective registries precludes a reliable and direct comparison, we addressed this limitation by strictly applying the same methodology to all registry analyses and selecting relevant, comparable variables to mitigate this bias. By nature, the observational design cannot lead to conclusion regarding causality. Additionally, there was a significant proportion of missing data on certain comorbidities, particularly BMI in the UKRR. Residual confounders which are known to impact home dialysis initiation (or KRT modality choice), such as the socio-economic status, the distance to the nearest dialysis unit, centre’s characteristics (such as pre-dialysis educational programmes, transit care units among others), were not fully captured in the registries. Although ethnicity is known to have an impact within individual countries, its classification and definition vary significantly across the countries included in this study. Therefore, we chose to exclude variables that were not consistently representative across all included countries. Despite these limitations, our findings appeared robust across sensitivity analyses [4, 5, 41].

In conclusion, our study highlights substantial international differences in home dialysis utilization and transition patterns over the first year of dialysis. Achieving broader uptake of home dialysis will likely require coordinate system-level strategies including transitional care models, assistance programmes, supportive healthcare policies and economic incentives.

## Supplementary Material

sfag271_Supplemental_File

## Data Availability

The CORR database is hosted by the Canadian Institute for Health Information, available via https://www.cihi.ca/en/canadian-organ-replacement-register-corr. The ANZDATA database is hosted by the Australia and New Zealand Organ Replacement Therapies Registry Group and its metadata are available via https://anzorrg.org.au/registries/anzdata. The REIN database is hosted by the Agence de la Biomédecine and its metadata are available via https://www.agence-biomedecine.fr/fr/observatoire-de-la-maladie-renale-chronique/le-registre-rein. The UKRR database is hosted by the UK Kidney Association and its metadata are available via https://www.ukkidney.org/about-us/who-we-are/uk-renal-registry. Individual-level data are not available for export. The data supporting the findings of this study are available from the corresponding author upon reasonable request.
