# Supplementary material for: International comparison of home dialysis uptake: a multi-registry analysis from the INTEGRATED Research Group
Source: Clin Kidney J. 2026 Aug 17;19(9):sfag271. doi: 10.1093/ckj/sfag271 (PMC13560579; doi:10.1093/ckj/sfag271)
Supplement: Supplementary file 1 [file sfag271_supplemental_file.docx]

**Supplementary data**

**Table S1.** Characteristics of each registry.

| **Variable** | | **ANZDATA** | **CORR** | **REIN** | **UKRR** |
| --- | --- | --- | --- | --- | --- |
| Available since | | 1977 | 1981 | 2002 | 1995 |
| Region covered | | Australia  & New Zealand | Canada | France | UK |
| Coverage | | 100% | 95%* | 100% | 99% |
| Reporting | | Yearly reports (general report, quality indicators report, additional special reports) | Yearly reports: general report, quality indicators | Yearly reports:  Supports public health decision-making and research | Yearly reports: general reports, live data portal |
| Patients included | | All patients receiving KRT | All patients receiving dialysis KRT | All patients receiving KRT | All patients receiving KRT |
| Definition of a transfer between dialysis modality | | All transfers between modalities are reported, irrespective of duration | All transfers between modalities are reported, irrespective of duration | At least 60 days in the new modality | Not routinely reported at time of analysis |
| Inclusion of pre-emptive transplant | | Yes, patients receiving pre-emptive transplants are reported to the registry in real-time | Patients with pre-emptive transplants are not systematically reported to the registry due to differences in provincial regulations | Yes, patients receiving pre-emptive transplants are reported to the registry in real-time | Yes, patients receiving pre-emptive transplants are reported to the registry |
|  |  |  |  |  |  |

*current CORR coverage is >95% although Quebec and Manitoba data were not included in the current study dataset with >80% coverage

**Table S2.** Characteristics of the patients experiencing home dialysis by registry according to their first home dialysis modality (PD or HHD) within the first year of dialysis initiation.

| **Variable** | **AUSTRALIA** | | **NZ** | | **CORR** | |
| --- | --- | --- | --- | --- | --- | --- |
|  | **PD (*n*=8,901)** | **HHD (*n*=1,344)** | **PD (*n*=2,589)** | **HHD (*n*=533)** | **PD (*n*=14,633)** | **HHD (*n*=1,442)** |
| First dialysis modality |  |  |  |  |  |  |
| In centre haemodialysis | 2,231 (25) | 223 (17) | 787 (30) | 474 (89) | 3,917 (27) | 1,127 (79) |
| Peritoneal dialysis | 6,670 (75) | 0 (0) | 1,802 (70) | 0 (0) | 10,716 (73) | 0 |
| Home haemodialysis | 0 (0) | 1,121 (83) | 0 (0) | 59 (11) | 0 | 315 (22) |
| Age at dialysis initiation, *median (IQR)* | 61 (49-71) | 54 (45-62) | 60 (50-68) | 51 (44-59) | 62 (52-72) | 57 (47-67) |
| Sex, male | 5,500 (62) | 994 (74) | 1,525 (59) | 392 (74) | 9,095 (62) | 963 (67) |
| Primary kidney disease |  |  |  |  |  |  |
| Diabetes | 3,086 (35) | 367 (28) | 1,241 (48) | 231 (43) | 5,776 (40) | 433 (30) |
| Glomerulonephritis | 2,152 (24) | 350 (26) | 553 (21) | 151 (28) | 2,469 (17) | 311 (22) |
| Vascular or hypertensive nephropathy | 1,311 (15)^a^ | 129 (10)^a^ | 290 (11)^a^ | 36 (7)^a^ | 3,222 (22) | 442 (31) |
| Other | 2,242 (26) | 475 (36) | 106 (4) | 115 (22) | 2,374 (16) | 157 (11) |
| Comorbidities |  |  |  |  |  |  |
| Diabetes | 4,050 (46) | 496 (37) | 1,392 (54) | 264 (50) | 7,466 (51) | 612 (42) |
| Pulmonary disease | 853 (10) | 103 (8) | 323 (13) | 68 (13) | 1,060 (7) | 94 (7) |
| Coronary disease | 2,536 (29) | 263 (20) | 642 (25) | 94 (18) | 2,080 (14) | 162 (11) |
| Peripheral vascular disease | 1,307 (15) | 125 (9) | 349 (14) | 30 (6) | 1,659 (11) | 123 (9) |
| Cerebrovascular disease | 871 (10) | 68 (5) | 273 (11) | 24 (5) | 1,459 (10) | 82 (6) |
| Malignancy | 721 (8)^b^ | 129 (10)^b^ | 212 (8)^b^ | 50 (9)^b^ | 1,475 (10) | 185 (13) |
| Current smoker | 1,017 (11) | 163 (12) | 390 (15) | 75 (14) | 1,940 (13) | 192 (13) |
| BMI |  |  |  |  |  |  |
| <20 Kg/m² | 603 (7) | 40 (3) | 95 (4) | 11 (2) | 1,051 (7) | 94 (7) |
| 20-24.9Kg/m² | 2,565 (29) | 255 (19) | 582 (23) | 66 (12) | 4,042 (28) | 296 (21) |
| 25-29.9 Kg/m² | 3,058 (35) | 348 (26) | 883 (34) | 113 (21) | 4,450 (30) | 358 (25) |
| 30 Kg/m² and over | 2,518 (29) | 673 (51) | 1,006 (39) | 341 (64) | 4,202 (29) | 547 (38) |
| Year of dialysis initiation |  |  |  |  |  |  |
| 2008-2011 | 3,173 (36) | 501 (37) | 997 (39) | 215 (40) | 4,334 (30) | 383 (27) |
| 2012-2015 | 3,804 (43) | 585 (44) | 1,025 (40) | 240 (45) | 5,394 (37) | 609 (42) |
| 2016-2018 | 1,924 (22)^c^ | 258 (19)^c^ | 567 (22)^c^ | 78 (15)^c^ | 4,905 (34) | 450 (31) |
| Results are expressed as absolute number (percentage), except for age at dialysis initiation reported as median (IQR).  NZ: New-Zealand, CORR: Canadian Organ Replacement Register, REIN: Réseau Epidémiologique et Information en Néphrologie, UKRR: United Kingdom Renal Registry, PD: peritoneal dialysis, HHD: home haemodialysis; BMI: body mass index, IQR: interquartile range a: hypertensive nephropathy; b: excluding skin cancer; c: 2016-2017*Probably linked to the declaration of HHD training in the registry | | | | | | |

| **Variable** | **REIN** | | **UKRR** | |
| --- | --- | --- | --- | --- |
|  | **PD (*n*=13,456)** | **HHD (*n*=274)** | **PD (*n*=18,072)** | **HHD (*n*=1,078)** |
| First dialysis modality |  |  |  |  |
| In centre haemodialysis | 2,189 (16) | 253 (92)* | 2,962 (16) | 966 (90) |
| Home haemodialysis | 0 (0) | 21 (8) | 11(1) | 112 (10) |
| Peritoneal dialysis | 11,267 (84) | 0 (0) | 15,099 (84) | 0 (0) |
| Age at dialysis initiation, *median (IQR)* | 71 (57-81) | 53 (42-63) | 60 (47-71) | 56 (47-66) |
| Sex, male | 8,236 (61) | 197 (72) | 11,381 (63) | 674 (63) |
| Primary kidney disease |  |  |  |  |
| Diabetes | 2,455 (18) | 20 (7) | 4,427 (25) | 220 (20) |
| Glomerulonephritis | 1,894 (14) | 76 (28) | 3,022 (17) | 181 (17) |
| Vascular or hypertensive nephropathy | 3,715 (28) | 36 (13) | 2,245 (12) | 67 (6) |
| Other | 5,392 (40) | 142 (52) | 7,976 (44) | 546 (51) |
| Comorbidities |  |  |  |  |
| Diabetes | 4,925 (37) | 47 (17) | 4,427 (25) | 220 (20) |
| Pulmonary disease | 1,575 (12) | 25 (9) | 552 (3) | 36 (3) |
| Coronary disease | 3,395 (25) | 28 (10) | 1,843 (10) | 72 (7) |
| Peripheral vascular disease | 2,395 (18) | 19 (7) | 1,076 (6) | 77 (7) |
| Cerebrovascular disease | 1,428 (11) | 11 (4) | 3,047 (17) | 142 (13) |
| Malignancy | 922 (7) | 34 (12) | 1,067 (6) | 87 (8) |
| Current smoker | 4,622 (34) | 110 (40) | 1,335 (7) | 61 (6) |
| BMI |  |  |  |  |
| <20 Kg/m² | 961 (7) | 20 (7) | 16 (0.1) | 0 (0) |
| 20-24.9Kg/m² | 4,079 (30) | 79 (29) | 64 (0.3) | 4 (0.3) |
| 25-29.9 Kg/m² | 3,790 (28) | 75 (27) | 78 (0.4) | 4 (0.3) |
| 30 Kg/m² and over | 1,995 (15) | 53 (19) | 54 (0.3) | 5 (0.4) |
| Year of dialysis initiation |  |  |  |  |
| 2008-2011 | 4,431 (33) | 35 (13) | 6,080 (34) | 315 (29) |
| 2012-2015 | 5,093 (38) | 101 (37) | 6,585 (36) | 389 (36) |
| 2016-2018 | 3,932 (29) | 138 (50) | 5,407 (30) | 374 (35) |
| Results are expressed as absolute number (percentage), except for age at dialysis initiation reported as median (IQR).  NZ: New-Zealand, CORR: Canadian Organ Replacement Register, REIN: Réseau Epidémiologique et Information en Néphrologie, UKRR: United Kingdom Renal Registry, PD: peritoneal dialysis, HHD: home haemodialysis; BMI: body mass index, IQR: interquartile range a: hypertensive nephropathy; b: excluding skin cancer; c: 2016-2017  *Probably linked to the declaration of HHD training in the registry | | | | |

**Table S3.** Patient’s characteristics by registry according to the timing of home dialysis initiation.

| **Variable** | **AUSTRALIA** | | **NZ** | | **CORR** | |
| --- | --- | --- | --- | --- | --- | --- |
|  | **Incident on home dialysis (*n*=6,893)** | **Transferred to home dialysis (*n*=3,352)** | **Incident on home dialysis (*n*=1,861)** | **Transferred to home dialysis (*n*=1,261)** | **Incident on home dialysis (*n*=11,031)** | **Transferred to home dialysis (*n*=5,044)** |
| Home dialysis modality |  |  |  |  |  |  |
| Peritoneal dialysis | 6,670 (97) | 2,231 (67) | 1,802 (97) | 787 (62) | 10,716 (97) | 3,917 (78) |
| Home haemodialysis | 223 (3) | 1,121 (33) | 59 (3) | 474 (38) | 315 (2.9) | 1,127 (22) |
| Age at dialysis initiation, *median (IQR)* | 62 (50-72) | 58 (47-67) | 60 (51-69) | 55 (46-64) | 62 (52-72) | 62 (47-72) |
| Sex, male | 4,247 (62) | 2,247 (67) | 1,064 (57) | 853 (68) | 6795 (62) | 3,263 (65) |
| Primary kidney disease |  |  |  |  |  |  |
| Diabetes | 2,338 (34) | 1,115 (34) | 882 (48) | 590 (47) | 4,362 (39) | 1,847 (37) |
| Glomerulonephritis | 1,627 (24) | 875 (26) | 389 (21) | 315 (25) | 1,872 (17) | 908 (18) |
| Vascular or hypertensive nephropathy | 1,018 (15)^a^ | 422 (13)^a^ | 224 (12)^a^ | 102 (8)^a^ | 1,833 (17) | 698 (14) |
| Other | 1,810 (27) | 907 (27) | 361 (19) | 250 (20) | 2,393 (22) | 1,271 (25) |
| Comorbidities |  |  |  |  |  |  |
| Diabetes | 3,092 (45) | 1,454 (43) | 997 (54) | 659 (52) | 5,539 (50) | 2,539 (50) |
| Pulmonary disease | 593 (9) | 363 (11) | 223 (12) | 168 (13) | 723 (6.6) | 431 (8.5) |
| Coronary disease | 1,852 (27) | 947 (28) | 432 (23) | 304 (24) | 1,417 (13) | 825 (16) |
| Peripheral vascular disease | 941 (14) | 491 (15) | 248 (13) | 131 (10) | 1,139 (10) | 643 (13) |
| Cerebrovascular disease | 669 (10) | 270 (8) | 195 (11) | 102 (8) | 1,026 (9.3) | 515 (10) |
| Malignancy | 530 (8)^b^ | 320 (10)^b^ | 150 (8)^b^ | 112 (9)^b^ | 1,063 (10) | 597 (12) |
| Current smoker | 769 (11) | 411 (12) | 263 (14) | 202 (16) | 1,367 (12) | 765 (15) |
| BMI |  |  |  |  |  |  |
| <20 Kg/m² | 177 (1) | 203 (6) | 24 (1) | 43 (3.5) | 785 (7) | 360 (7) |
| 20-24.9Kg/m² | 2,211 (32) | 872 (26) | 444 (24) | 243 (19) | 3,087 (28) | 1,251 (25) |
| 25-29.9 Kg/m² | 2,375 (35) | 1,031 (31) | 644 (35) | 352 (28) | 3,398 (31) | 1,410 (28) |
| 30 Kg/m² and over | 2,002 (30) | 1,189 (35) | 730 (39) | 617 (49) | 3,178 (29) | 1,571 (31) |
| Year of dialysis initiation |  |  |  |  |  |  |
| 2008-2011 | 2,239 (33) | 1,435 (43) | 656 (35) | 556 (44) | 3,228 (29) | 1,489 (29) |
| 2012-2015 | 2,987 (43) | 1,402 (42) | 766 (41) | 499 (40) | 3,996 (36) | 2,007 (40) |
| 2016-2018 | 1,667 (24)^c^ | 515 (15)^c^ | 439 (24)^c^ | 206 (16)^c^ | 3,807 (35) | 1,548 (31) |
| Results are expressed as absolute number (percentage), except for age at dialysis initiation reported as median (IQR).  NZ: New-Zealand, CORR: Canadian Organ Replacement Register, REIN: Réseau Epidémiologique et Information en Néphrologie, UKRR: United Kingdom Renal Registry; BMI: body mass index, IQR: interquartile range  a: hypertensive nephropathy; b: excluding skin cancer; c: 2016-2017 | | | | | | |

| **Variable** | **REIN** | | **UKRR** | |
| --- | --- | --- | --- | --- |
|  | **Incident on home dialysis (*n*= 11,288)** | **Transferred to home dialysis (*n*=2,442)** | **Incident on home dialysis (*n*=15,222)** | **Transferred to home dialysis (*n*=3,928)** |
| Home dialysis modality |  |  |  |  |
| Peritoneal dialysis | 11,267 (99.8) | 2,189 (90) | 15,099 (21) | 2,962 (75.4) |
| Home haemodialysis | 21 (0.2) | 253 (10) | 123 (0,2) | 966 (24.6) |
| Age at dialysis initiation, *median (IQR)* | 72 (58-81) | 66 (50-79) | 60 (47-71) | 58 (45-69) |
| Sex, male | 6,842 (61) | 1,591 (65) | 9,532 (63) | 2,523 (64) |
| Primary kidney disease |  |  |  |  |
| Diabetes | 2,087 (18) | 388 (16) | 3,746 (25) | 901 (23) |
| Glomerulonephritis | 1,566 (14) | 404 (17) | 2,552 (17) | 651 (16) |
| Vascular or hypertensive nephropathy | 3,160 (28) | 591 (24) | 1,883 (12) | 429 (11) |
| Other | 4,475 (40) | 1,059 (43) | 6,728 (44) | 1,794 (46) |
| Comorbidities |  |  |  |  |
| Diabetes | 4,153 (37) | 819 (34) | 666 (4) | 198 (8) |
| Pulmonary disease | 1,323 (12) | 277 (11) | 444 (3) | 144 (6) |
| Coronary disease | 2,811 (25) | 612 (25) | 1,526 (10) | 389 (17) |
| Peripheral vascular disease | 2,017 (18) | 397 (16) | 882 (6) | 271 (11) |
| Cerebrovascular disease | 1,195 (11) | 244 (10) | 2,528 (17) | 661 (29) |
| Malignancy | 752 (7) | 204 (8) | 871 (6) | 283 (12) |
| Current smoker | 3,829 (34) | 903 (37) | 1,089 (7) | 307 (14) |
| BMI |  |  |  |  |
| <20 Kg/m² | 752 (7) | 229 (9) | 14 (0) | 2 (0) |
| 20-24.9Kg/m² | 3,322 (29) | 836 (34) | 55 (0) | 13 (0) |
| 25-29.9 Kg/m² | 3,243 (29) | 622 (26) | 64 (0) | 17 (0) |
| 30 Kg/m² and over | 1,737 (15) | 311 (13) | 49 (0) | 10 (0) |
| Year of dialysis initiation |  |  |  |  |
| 2008-2011 | 3,705 (33) | 761 (31) | 5,072 (33) | 1,323 (34) |
| 2012-2015 | 4,268 (38) | 926 (38) | 5,561 (37) | 1,414 (36) |
| 2016-2018 | 3,315 (29) | 755 (31) | 4,589 (30) | 1,192 (30) |
| Results are expressed as absolute number (percentage), except for age at dialysis initiation reported as median (IQR).  NZ: New-Zealand, CORR: Canadian Organ Replacement Register, REIN: Réseau Epidémiologique et Information en Néphrologie, UKRR: United Kingdom Renal Registry; BMI: body mass index, IQR: interquartile range  a: hypertensive nephropathy; b: excluding skin cancer; c: 2016-2017 | | | | |

**Table S4.** Sensitivity analysis: Adjusted clustered Cox model of home dialysis initiation within one year after dialysis initiation, excluding variables with significant missingness.

| **Explanatory variable** | | **AUSTRALIA** | **NZ** | **CORR** | **REIN** | **UKRR** |
| --- | --- | --- | --- | --- | --- | --- |
|  |  | **HR (95% CI)** | **HR (95% CI)** | **HR (95% CI)** | **HR (95% CI)** | **HR (95% CI)** |
| Interval in month between dialysis and home dialysis initiation | |  |  |  |  |  |
| Month 1 | | 12.0 (11.1-13.0) | 7.97 (7.00-9.07) | 11.5 (10.7-12.3) | 15.5 (14.3-16.7) | 27.7 (25.5-30.2) |
| Month 2 | | reference | reference | reference | reference | reference |
| Month 3 | | 0.96 (0.86-1.07) | 0.78 (0.65-0.94) | 1.07 (0.97-1.19) | 0.77 (0.69-0.87) | 0.83 (0.73-0.93) |
| Month 4 | | 0.72 (0.63-0.81) | 0.56 (0.46-0.68) | 0.91 (0.82-1.01) | 0.42 (0.36-0.48) | 0.65 (0.57-0.74) |
| Month 5 | | 0.52 (0.46-0.60) | 0.43 (0.35-0.54) | 0.90 (0.81-1.00) | 0.35 (0.31-0.41) | 0.65 (0.57-0.74) |
| Month 6 | | 0.38 (0.33-0.44) | 0.26 (0.20-0.34) | 0.68 (0.61-0.77) | 0.16 (0.13-0.20) | 0.56 (0.48-0.63) |
| Month 7 | | 0.30 (0.26-0.36) | 0.26 (0.20-0.34) | 0.52 (0.45-0.59) | 0.13 (0.10-0.16) | 0.39 (0.34-0.46) |
| Month 8 | | 0.23 (0.19-0.27) | 0.19 (0.14-0.26) | 0.46 (0.40-0.53) | 0.14 (0.11-0.18) | 0.35 (0.30-0.42) |
| Month 9 | | 0.14 (0.11-0.17) | 0.22 (0.16-0.29) | 0.36 (0.30-0.42) | 0.11 (0.08-0.14) | 0.27 (0.23-0.32) |
| Month 10 | | 0.12 (0.10-0.15) | 0.14 (0.10-0.20) | 0.33 (0.28-0.38) | 0.10 (0.07-0.13) | 0.28 (0.23-0.33) |
| Month 11 | | 0.11 (0.09-0.14) | 0.13 (0.09-0.18) | 0.28 (0.24-0.33) | 0.07 (0.05-0.10) | 0.22 (0.18-0.26) |
| Month 12 | | 0.12 (0.09-0.15) | 0.07 (0.04-0.11) | 0.14 (0.10-0.21) | 0.10 (0.08-0.14) | 0.20 (0.17-0.25) |
| Age at dialysis initiation | |  |  |  |  |  |
| <50 | | reference | reference | reference | reference | reference |
| 50-65 | | 0.90 (0.86-0.93) | 0.96 (0.89-1.03) | 0.85 (0.81-0.88) | 0.68 (0.64-0.71) | 0.75 (0.72-0.77) |
| 66-75 | | 0.75 (0.71-0.78) | 0.92 (0.84-1.00) | 0.69 (0.66-0.72) | 0.60 (0.57-0.64) | 0.58 (0.56-0.60) |
| >75 | | 0.54 (0.51-0.57) | 0.70 (0.61-0.81) | 0.5 (0.48-0.52) | 0.71 (0.68-0.74) | 0.42 (0.40-0.44) |
| Female sex | | 0.93 (0.89-0.96) | 0.97 (0.91-1.04) | 0.96 (0.93 -0.99) | 1.12 (1.09-1.17) | 1.00 (0.98-1.02) |
| Primary kidney disease | |  |  |  |  |  |
| Diabetes | | reference | reference | reference | reference | reference |
| Glomerulonephritis | | 1.41 (1.35-1.48) | 1.34 (1.23-1.45) | 1.32 (1.26-1.38) | 1.55 (1.46-1.64) | 1.41 (1.36-1.47) |
| Vascular or hypertensive nephropathy | | 1.27 (1.20-1.35)^a^ | 1.34 (1.20-1.50) | 1.13 (1.08-1.19) | 1.25 (1.19-1.32) | 1.18 (1.13-1.23) |
| Other | | 1.15 (1.10-1.20) | 1.19 (1.10-1.30) | 0.84 (0.81-0.88) | 1.21 (1.15-1.27) | 1.11 (0.75-0.89) |
| Year of dialysis initiation | |  |  |  |  |  |
| 2008-2011 | | reference | reference | reference | reference | reference |
| 2012-2015 | | 1.11 (1.07-1.15) | 1.01 (0.94-1.08) | 1.16 (1.12-1.21) | 1.01 (0.97-1.06) | 1.03 (1.00-1.06) |
| 2016-2018 | | 0.99 (0.94-1.04)^b^ | 0.97 (0.89-1.06) | 1.29 (1.24-1.34) | 1.00 (0.97-1.05) | 1.05 (1.02-1.08) |
| NZ: New-Zealand, CORR: Canadian Organ Replacement Register, REIN: Réseau Epidémiologique et Information en Néphrologie, UKRR: United Kingdom Renal Registry,  a: hypertensive nephropathy; b: 2016-2017 | | | | | | |
|  |  |  |  |  |  |  |

**Table S5.** Adjusted clustered Cox model of peritoneal dialysis initiation within one year after dialysis initiation.

| **Explanatory variable** | | **AUSTRALIA** | **NZ** | **CORR** | **REIN** | **UKRR** |
| --- | --- | --- | --- | --- | --- | --- |
|  |  | **HR (95% CI)** | **HR (95% CI)** | **HR (95% CI)** | **HR (95% CI)** | **HR (95% CI)** |
| Interval in month between dialysis and home dialysis initiation | |  |  |  |  |  |
| Month 1 | | 15.0 (13.7-16.5) | 9.24 (8.00-10.7) | 13.8 (12.6-15.1) | 16.2 (14.7-17.9) | 31.3 (28.7-34.2) |
| Month 2 | | reference | reference | reference | reference | reference |
| Month 3 | | 0.81 (0.71-0.93) | 0.71 (0.58-0.88) | 1.01 (0.89-1.15) | 0.82 (0.72-0.95) | 0.74 (0.65-0.85) |
| Month 4 | | 0.62 (0.54-0.72) | 0.42 (0.33-0.54) | 0.85 (0.74-0.97) | 0.40 (0.33-0.48) | 0.54 (0.46-0.62) |
| Month 5 | | 0.40 (0.34-0.48) | 0.30 (0.22-0.40) | 0.87 (0.76-0.99) | 0.33 (0.27-0.40) | 0.52 (0.45-0.61) |
| Month 6 | | 0.29 (0.24-0.36) | 0.13 (0.09-0.19) | 0.69 (0.60-0.80) | 0.15 (0.11-0.20) | 0.46 (0.39-0.53) |
| Month 7 | | 0.24 (0.20-0.30) | 0.12 (0.08-0.18) | 0.54 (0.46-0.63) | 0.12 (0.09-0.16) | 0.27 (0.22-0.33) |
| Month 8 | | 0.14 (0.11-0.18) | 0.10 (0.06-0.16) | 0.38 (0.32-0.46) | 0.11 (0.08-0.15) | 0.25 (0.21-0.30) |
| Month 9 | | 0.09 (0.07-0.13) | 0.13 (0.09-0.19) | 0.31 (0.26-0.38) | 0.07 (0.05-0.10) | 0.18 (0.14-0.22) |
| Month 10 | | 0.08 (0.06-0.11) | 0.05 (0.03-0.10) | 0.30 (0.24-0.37) | 0.07 (0.05-0.10) | 0.15 (0.11-0.19) |
| Month 11 | | 0.08 (0.06-0.11) | 0.06 (0.03-0.10) | 0.28 (0.22-0.35) | 0.05 (0.03-0.08) | 0.12 (0.09-0.16) |
| Month 12 | | 0.07 (0.05-0.10) | 0.03 (0.01-0.06) | 0.14 (0.09-0.22) | 0.07 (0.05-0.10) | 0.12 (0.09-0.16) |
| Age at dialysis initiation | |  |  |  |  |  |
| <50 | | reference | reference | reference | reference | reference |
| 50-65 | | 0.95 (0.90-1.00) | 1.14 (1.05-1.25) | 0.97 (0.93-1.02) | 0.72 (0.67-0.77) | 0.76 (0.73-0.79) |
| 65-75 | | 0.92 (0.87-0.97) | 1.20 (1.09-1.33) | 0.87 (0.83-0.92) | 0.65 (0.60-0.70) | 0.61 (0.59-0.64) |
| ≥75 | | 0.69 (0.65-0.74) | 0.94 (0.80-1.10) | 0.63 (0.60-0.67) | 0.73 (0.69-0.78) | 0.46 (0.44-0.48) |
| Female sex | | 1.00 (0.96-1.04) | 1.13 (1.06-1.21) | 0.98 (0.94-1.01) | 1.17 (1.12-1.23) | 1.00 (0.97-1.03) |
| Primary kidney disease | |  |  |  |  |  |
| Diabetes | | reference | reference | reference | reference | reference |
| Glomerulonephritis | | 1.12 (1.04-1.20) | 0.88 (0.75-1.04) | 0.95 (0.88-1.01) | 1.35 (1.23-1.47) | 1.41 (1.36-1.47) |
| Vascular or hypertensive nephropathy | | 1.06 (0.98-1.15)^a^ | 0.95 (0.80-1.14)^a^ | 0.92 (0.85-0.98) | 1.06 (0.98-1.15) | 1.22 (1.16-1.28) |
| Other | | 0.89 (0.83-0.96) | 0.81 (0.69-0.96) | 0.67 (0.63-0.71) | 1.07 (0.99-1.15) | 1.20 (1.07-1.14) |
| BMI | |  |  |  |  |  |
| <20 Kg/m² | | 0.95 (0.87-1.02) | 0.88 (0.73-1.05) | 0.89 (0.83-0.96) | 0.76 (0.70-0.82) | 0.93 (0.57-1.50) |
| 20-24.9Kg/m² | | reference | reference | reference | reference | reference |
| 25-29.9 Kg/m² | | 1.00 (0.96-1.05) | 0.96 (0.88-1.05) | 1.02 (0.98-1.07) | 1.08 (1.03-1.14) | 1.07 (0.80-1.44) |
| 30 Kg/m² and over | | 0.72 (0.68-0.75) | 0.61 (0.56-0.67) | 0.83 (0.80-0.87) | 0.77 (0.73-0.82) | 0.69 (0.49-0.95) |
| Comorbidities | |  |  |  |  |  |
| Diabetes | | 0.89 (0.83-0.95) | 0.75 (0.65-0.88) | 0.80 (0.75-0.85) | 0.85 (0.80-0.90) | 0.78 (0.73-0.84) |
| Pulmonary disease | | 0.78 (0.73-0.83) | 0.81 (0.73-0.90) | 0.67 (0.62-0.72) | 0.79 (0.74-0.84) | 0.76 (0.70-0.82) |
| Coronary disease | | 0.89 (0.85-0.94) | 0.89 (0.82-0.97) | 0.84 (0.8-0.89) | 1.12 (1.06-1.18) | 1.04 (0.97-1.11) |
| Peripheral vascular disease | | 0.88 (0.83-0.94) | 1.03 (0.92-1.14) | 0.79 (0.75-0.84) | 0.96 (0.90-1.02) | 0.88 (0.82-0.95) |
| Cerebrovascular disease | | 0.95 (0.89-1.02) | 0.97 (0.87-1.09) | 0.82 (0.77-0.87) | 1.01 (0.95-1.09) | 1.02 (0.96-1.09) |
| Malignancy | | 0.72 (0.67-0.77)^b^ | 0.85 (0.75-0.97)^b^ | 0.76 (0.72-0.80) | 0.61 (0.56-0.66) | 0.86 (0.80-0.91) |
| Current smoker | | 0.84 (0.79-0.90) | 0.95 (0.86-1.04) | 0.84 (0.80-0.89) | 1.05 (1-1.10) | 1.02 (0.97-1.07) |
| Year of dialysis initiation | |  |  |  |  |  |
| 2008-2011 | | reference | reference |  | reference | reference |
| 2012-2015 | | 1.11 (1.07-1.16) | 1.02 (0.95-1.10) | 1.09 (1.04-1.14) | 1 (0.95-1.05) | 1.03 (0.99-1.06) |
| 2016-2018 | | 1.03 (0.97-1.08)^c^ | 1.08 (0.99-1.18)^c^ | 1.24 (1.19-1.29) | 0.92 (0.87-0.97) | 1.04 (1.00-1.07) |
| NZ: New-Zealand, CORR: Canadian Organ Replacement Register, REIN: Réseau Epidémiologique et Information en Néphrologie, UKRR: United Kingdom Renal Registry, BMI: body mass index  a: hypertensive nephropathy; b: excluding skin cancer; c: 2016-2017 | | | | | | |
|  |  |  |  |  |  |  |

**Table S6.** Sensitivity analysis: Adjusted clustered Cox model of peritoneal dialysis initiation within one year after dialysis initiation, excluding variables with significant missingness.

| **Explanatory variable** | | **AUSTRALIA** | **NZ** | **CORR** | **REIN** | **UKRR** |
| --- | --- | --- | --- | --- | --- | --- |
|  |  | **HR (95% CI)** | **HR (95% CI)** | **HR (95% CI)** | **HR (95% CI)** | **HR (95% CI)** |
| Interval in month between dialysis and home dialysis initiation | |  |  |  |  |  |
| Month 1 | | 14.9 (13.6-16.4) | 9.24 (8.01-10.7) | 13.4 (12.4-14.5) | 15.7 (14.6-17.0) | 31.3 (28.8-34.2) |
| Month 2 | | reference | reference | reference | reference | reference |
| Month 3 | | 0.81 (0.70-0.92) | 0.71 (0.58-0.88) | 1.04 (0.93-1.17) | 0.76 (0.68-0.85) | 0.74 (0.65-0.85) |
| Month 4 | | 0.61 (0.53-0.71) | 0.42 (0.33-0.53) | 0.85 (0.76-0.96) | 0.38 (0.32-0.43) | 0.54 (0.46-0.62) |
| Month 5 | | 0.40 (0.34-0.47) | 0.29 (0.22-0.39) | 0.90 (0.79-1.01) | 0.30 (0.26-0.35) | 0.52 (0.45-0.61) |
| Month 6 | | 0.29 (0.24-0.35) | 0.13 (0.09-0.19) | 0.69 (0.60- 0.79) | 0.13 (0.10-0.16) | 0.46 (0.39-0.53) |
| Month 7 | | 0.24 (0.20-0.30) | 0.12 (0.07-0.18) | 0.54 (0.47-0.63) | 0.10 (0.07-0.13) | 0.27 (0.22-0.32) |
| Month 8 | | 0.14 (0.11-0.18) | 0.10 (0.06-0.16) | 0.41 (0.35-0.48) | 0.10 (0.08-0.13) | 0.25 (0.21-0.30) |
| Month 9 | | 0.09 (0.07-0.12) | 0.13 (0.09-0.19) | 0.31 (0.26-0.37) | 0.07 (0.05-0.09) | 0.17 (0.14-0.22) |
| Month 10 | | 0.08 (0.06-0.11) | 0.05 (0.03-0.10) | 0.30 (0.25-0.36) | 0.07 (0.05-0.10) | 0.15 (0.11-0.19) |
| Month 11 | | 0.08 (0.06-0.11) | 0.06 (0.03-0.10) | 0.26 (0.22-0.32) | 0.05 (0.03-0.07) | 0.12 (0.09-0.16) |
| Month 12 | | 0.07 (0.05-0.10) | 0.03 (0.01-0.06) | 0.14 (0.10-0.21) | 0.06 (0.04-0.09) | 0.12 (0.09-0.16) |
| Age at dialysis initiation | |  |  |  |  |  |
| <50 | | reference | reference | reference | reference | reference |
| 50-65 | | 0.89 (0.85-0.93) | 1.08 (0.99-1.17) | 0.88 (0.84-0.92) | 0.69 (0.65-0.73) | 0.74 (0.72-0.77) |
| 66-75 | | 0.84 (0.80-0.89) | 1.14 (1.04-1.26) | 0.75 (0.72-0.79) | 0.62 (0.65-0.73) | 0.59 (0.57-0.61) |
| >75 | | 0.65 (0.61-0.69) | 0.95 (0.82-1.10) | 0.57 (0.54-0.60) | 0.75 (0.71-0.78) | 0.44 (0.42-0.46) |
| Female sex | | 1.00 (0.97-1.04) | 1.08 (1.01-1.16) | 0.98 (0.95-1.01) | 1.14 (1.10-1.18) | 1.00 (0.98-1.03) |
| Primary kidney disease | |  |  |  |  |  |
| Diabetes | | reference | reference | reference | reference | reference |
| Glomerulonephritis | | 1.35 (1.29-1.42) | 1.25 (1.14-1.36) | 1.27 (1.22-1.33) | 1.51 (1.43-1.61) | 1.39 (1.34-1.45) |
| Vascular or hypertensive nephropathy | | 1.23 (1.16-1.31)^a^ | 1.31 (1.17-1.47)^a^ | 1.12 (1.07-1.17) | 1.24 (1.18-1.31) | 1.18 (1.13-1.24) |
| Other | | 1.04 (0.99-1.09) | 1.13 (1.03-1.24) | 0.79 (0.76-0.83) | 1.19 (1.13-1.25) | 1.07 (1.04-1.11) |
| Year of dialysis initiation | |  |  |  |  |  |
| 2008-2011 | | reference | reference | reference | reference | reference |
| 2012-2015 | | 1.11 (1.07-1.16) | 0.99 (0.92-1.06) | 1.14 (1.09-1.18) | 0.99 (0.96-1.04) | 1.02 (0.99-1.06) |
| 2016-2018 | | 1.02 (0.97-1.07)^b^ | 1.03 (0.94-1.12)^b^ | 1.28 (1.23-1.33) | 0.98 (0.94-1.02) | 1.03 (1.00-1.07) |
| NZ: New-Zealand, CORR: Canadian Organ Replacement Register, REIN: Réseau Epidémiologique et Information en Néphrologie, UKRR: United Kingdom Renal Registry, BMI: body mass index  a: hypertensive nephropathy; b: 2016-2017 | | | | | | |
|  |  |  |  |  |  |  |

**Table S7.** Adjusted clustered Cox model of home haemodialysis initiation within one year after dialysis initiation.

| **Explanatory variable** | | **AUSTRALIA** | **NZ** | **CORR** | **REIN** | **UKRR** |
| --- | --- | --- | --- | --- | --- | --- |
|  |  | **HR (95% CI)** | **HR (95% CI)** | **HR (95% CI)** | **HR (95% CI)** | **HR (95% CI)** |
| Interval in month between dialysis and home dialysis initiation | |  |  |  |  |  |
| Month 1 | | 2.09 (1.71-2.55) | 2.47 (1.77-3.44) | 2.88 (2.33-3.55) | 1.59 (0.73-3.47) | 2.22 (1.68-2.94) |
| Month 2 | | reference | reference | reference | reference | reference |
| Month 3 | | 1.46 (1.18-1.81) | 1.10 (0.75-1.62) | 1.17 (0.91-1.52) | 1.74 (0.80-3.81) | 1.42 (1.05-1.92) |
| Month 4 | | 1.08 (0.86-1.36) | 1.16 (0.79-1.70) | 1.22 (0.94-1.58) | 2.41 (1.15-5.07) | 1.48 (1.09-1.99) |
| Month 5 | | 0.95 (0.75-1.20) | 1.02 (0.69-1.51) | 1.08 (0.82-1.41) | 2.57 (1.23-5.38) | 1.59 (1.18-2.14) |
| Month 6 | | 0.69 (0.53-0.90) | 0.84 (0.55-1.27) | 0.67 (0.49-0.92) | 1.75 (0.79-3.85) | 1.27 (0.92-1.73) |
| Month 7 | | 0.50 (0.38-0.67) | 0.88 (0.58-1.32) | 0.45 (0.31-0.64) | 1.90 (0.87-4.14) | 1.31 (0.96-1.78) |
| Month 8 | | 0.53 (0.40-0.70) | 0.57 (0.36-0.91) | 0.68 (0.50-0.94) | 2.73 (1.31-5.71) | 1.09 (0.79-1.52) |
| Month 9 | | 0.29 (0.20-0.41) | 0.61 (0.39-0.96) | 0.59 (0.42-0.83) | 2.09 (0.96-4.52) | 0.92 (0.66-1.29) |
| Month 10 | | 0.25 (0.17-0.36) | 0.51 (0.32-0.83) | 0.49 (0.34-0.70) | 1.54 (0.68-3.51) | 1.19 (0.87-1.64) |
| Month 11 | | 0.21 (0.14-0.31) | 0.43 (0.26-0.71) | 0.31 (0.20-0.48) | 1.21 (0.50-2.90) | 0.88 (0.63-1.25) |
| Month 12 | | 0.26 (0.18-0.37) | 0.24 (0.13-0.46) | 0.15 (0.06-0.38) | 1.47 (0.65-3.33) | 0.77 (0.54-1.10) |
| Age at dialysis initiation | |  |  |  |  |  |
| <50 | | reference | reference | reference | Reference | reference |
| 50-65 | | 0.93 (0.82-1.05) | 0.64 (0.53-0.79) | 0.65 (0.57-0.75) | 0.47 (0.34-0.66) | 0.79 (0.68-0.91) |
| 66-75 | | 0.34 (0.28-0.41) | 0.31 (0.23-0.42) | 0.32 (0.27-0.39) | 0.21 (0.13-0.33) | 0.43 (0.36-0.50) |
| >75 | | 0.08 (0.06-0.12) | 0.08 (0.03-0.19) | 0.11 (0.09-0.15) | 0.08 (0.05-0.14) | 0.15 (0.12-0.19) |
| Female sex | | 0.51 (0.45-0.58) | 0.57 (0.47-0.69) | 0.77 (0.68-0.88) | 0.79 (0.58-1.08) | 0.99 (0.88-1.13) |
| Primary kidney disease | |  |  |  |  |  |
| Diabetes | | reference | reference | reference | reference | reference |
| Glomerulonephritis | | 1.26 (1.01-1.57) | 1.27 (0.85-1.88) | 1.40 (1.11-1.77) | 1.90 (0.90-3.98) | 1.95 (1.59-2.39) |
| Vascular or hypertensive nephropathy | | 1.15 (0.89-1.49)^a^ | 0.96 (0.59-1.58)^a^ | 1.10 (0.84-1.43) | 0.73 (0.32-1.66) | 0.92 (0.70-1.21) |
| Other | | 1.47 (1.19-1.82) | 1.12 (0.75-1.66) | 1.20 (0.97-1.49) | 1.25 (0.60-2.57) | 1.68 (1.43-1.98) |
| BMI | |  |  |  |  |  |
| <20 Kg/m² | | 0.60 (0.43-0.85) | 0.74 (0.39-1.41) | 1.09 (0.85-1.40) | 0.64 (0.37-1.08) | NA* |
| 20-24.9Kg/m² | | reference | reference | reference | reference | reference |
| 25-29.9 Kg/m² | | 1.22 (1.04-1.44) | 1.15 (0.85-1.56) | 1.21 (1.02-1.43) | 1.23 (0.85-1.80) | 0.84 (0.21-3.34) |
| 30 Kg/m² and over | | 1.94 (1.67-2.24) | 1.61 (1.23-2.10) | 1.52 (1.30-1.78) | 1.33 (0.95-1.87) | 0.83 (0.22-3.08) |
| Comorbidities | |  |  |  |  |  |
| Diabetes | | 0.64 (0.53-0.77) | 0.69 (0.47-1.00) | 0.74 (0.60-0.90) | 0.38 (0.22-0.65) | 0.91 (0.69-1.22) |
| Pulmonary disease | | 0.74 (0.60-0.91) | 0.85 (0.65-1.10) | 0.67 (0.52-0.86) | 0.98 (0.60-1.61) | 1.01 (0.72-1.42) |
| Coronary disease | | 0.72 (0.62-0.83) | 0.85 (0.67-1.08) | 0.88 (0.73-1.06) | 0.63 (0.37-1.08) | 0.96 (0.68-1.34) |
| Peripheral vascular disease | | 0.68 (0.56-0.83) | 0.52 (0.35-0.76) | 0.73 (0.59-0.90) | 0.83 (0.45-1.52) | 1.92 (1.38-2.68) |
| Cerebrovascular disease | | 0.66 (0.52-0.85) | 0.56 (0.37-0.85) | 0.57 (0.44-0.74) | 0.61 (0.30-1.25) | 0.69 (0.49-0.95) |
| Malignancy | | 0.90 (0.75-1.09)^b^ | 1.30 (0.95-1.79)^b^ | 1.21 (1.01-1.46) | 1.60 (1.04-2.44) | 1.44 (1.13-1.83) |
| Current smoker | | 0.71 (0.60-0.84) | 0.67 (0.52-0.85) | 0.61 (0.51-0.73) | 1.11 (0.83-1.48) | 0.77 (0.59-1.00) |
| Year of dialysis initiation | |  |  |  |  |  |
| 2008-2011 | | reference | reference |  | reference | reference |
| 2012-2015 | | 1.10 (0.98-1.24) | 1.11 (0.92-1.33) | 1.39 (1.20-1.62) | 2.63 (1.64-4.21) | 1.17 (1.02-1.37) |
| 2016-2018 | | 0.80 (0.68-0.93)^c^ | 0.75 (0.57-0.97)^c^ | 1.29 (1.11-1.51) | 5.01 (3.19-7.86) | 1.31 (1.12-1.52) |
| NZ: New-Zealand, CORR: Canadian Organ Replacement Register, REIN: Réseau Epidémiologique et Information en Néphrologie, UKRR: United Kingdom Renal Registry, BMI: body mass index  a: hypertensive nephropathy; b: excluding skin cancer; c: 2016-2017 | | | | | | |
|  |  |  |  |  |  |  |

**Table S8.** Sensitivity analysis: Adjusted clustered Cox model of home haemodialysis initiation within one year after dialysis initiation, excluding variables with significant missingness.

| **Explanatory variable** | | **AUSTRALIA** | **NZ** | **CORR** | **REIN** | **UKRR** |
| --- | --- | --- | --- | --- | --- | --- |
|  |  | **HR (95% CI)** | **HR (95% CI)** | **HR (95% CI)** | **HR (95% CI)** | **HR (95% CI)** |
| Interval in month between dialysis and home dialysis initiation | |  |  |  |  |  |
| Month 1 | | 2.20 (1.81-2.68) | 2.51 (1.80-3.50) | 2.72 (2.25-3.28) | 1.54 (0.79-2.99) | 2.22 (1.68-2.94) |
| Month 2 | | reference | reference | reference | reference | reference |
| Month 3 | | 1.47 (1.19-1.82) | 1.10 (0.75-1.62) | 1.21 (0.96-1.52) | 1.47 (0.74-2.91) | 1.42 (1.05-1.92) |
| Month 4 | | 1.06 (0.84-1.33) | 1.16 (0.79-1.70) | 1.13 (0.90-1.43) | 2.41 (1.28-4.51) | 1.48 (1.09-1.99) |
| Month 5 | | 0.93 (0.73-1.18) | 1.02 (0.69-1.51) | 0.93 (0.72-1.19) | 2.92 (1.58-5.39) | 1.59 (1.18-2.14) |
| Month 6 | | 0.68 (0.52-0.88) | 0.84 (0.55-1.27) | 0.66 (0.50-0.87) | 1.72 (0.88-3.37) | 1.27 (0.92-1.73) |
| Month 7 | | 0.50 (0.38-0.67) | 0.88 (0.58-1.32) | 0.41 (0.29-0.57) | 1.60 (0.81-3.17) | 1.31 (0.96-1.78) |
| Month 8 | | 0.52 (0.40-0.69) | 0.57 (0.36-0.91) | 0.71 (0.54-0.94) | 2.20 (1.15-4.20) | 1.09 (0.79-1.52) |
| Month 9 | | 0.29 (0.20-0.41) | 0.61 (0.39-0.96) | 0.55 (0.40-0.75) | 1.99 (1.03-3.85) | 0.92 (0.66-1.29) |
| Month 10 | | 0.27 (0.19-0.38) | 0.51 (0.32-0.83) | 0.46 (0.33-0.64) | 1.35 (0.66-2.78) | 1.19 (0.87-1.64) |
| Month 11 | | 0.22 (0.15-0.32) | 0.43 (0.26-0.71) | 0.36 (0.25-0.51) | 1.21 (0.58-2.53) | 0.88 (0.63-1.25) |
| Month 12 | | 0.27 (0.19-0.38) | 0.24 (0.13-0.46) | 0.16 (0.07-0.35) | 1.93 (1.00-3.72) | 0.77 (0.54-1.10) |
| Age at dialysis initiation | |  |  |  |  |  |
| <50 | | reference | reference | reference | reference | reference |
| 50-65 | | 0.92 (0.81-1.03) | 0.64 (0.53-0.78) | 0.63 (0.56-0.72) | 0.45 (0.34-0.59) | 0.79 (0.68-0.91) |
| 66-75 | | 0.31 (0.26-0.37) | 0.30 (0.23-0.40) | 0.31 (0.27-0.36) | 0.18 (0.12-0.26) | 0.43 (0.37-0.51) |
| >75 | | 0.07 (0.05-0.10) | 0.06 (0.03-0.15) | 0.11 (0.09-0.14) | 0.06 (0.04-0.09) | 0.15 (0.12-0.20) |
| Female sex | | 0.54 (0.48-0.61) | 0.57 (0.47-0.69) | 0.79 (0.70-0.88) | 0.70 (0.53-0.91) | 0.99 (0.88-1.12) |
| Primary kidney disease | |  |  |  |  |  |
| Diabetes | | reference | reference | reference | Reference | reference |
| Glomerulonephritis | | 1.98 (1.71-2.29) | 1.81 (1.46-2.24) | 1.91 (1.64-2.21) | 5.03 (3.04-8.32) | 1.91 (1.56-2.32) |
| Vascular or hypertensive nephropathy | | 1.54 (1.25-1.88)^a^ | 1.39 (0.97-1.98)^a^ | 1.31 (1.09-1.58) | 2.10 (1.20-3.65) | 0.89 (0.68-1.17) |
| Other | | 2.14 (1.87-2.45) | 1.51 (1.20-1.91) | 1.52 (1.33-1.74) | 3.46 (2.16-5.55) | 1.68 (1.43-1.97) |
| Year of dialysis initiation | |  |  |  |  |  |
| 2008-2011 | | reference | reference | reference | reference | reference |
| 2012-2015 | | 1.10 (0.98-1.24) | 1.10 (0.92-1.33) | 1.45 (1.27-1.65) | 2.60 (1.77-3.82) | 1.17 (1.01-1.37) |
| 2016-2018 | | 0.84 (0.72-0.97)^b^ | 0.72 (0.56-0.94)^b^ | 1.33 (1.16-1.53) | 4.74 (3.27-6.87) | 1.33 (1.14-1.54) |
| NZ: New-Zealand, CORR: Canadian Organ Replacement Register, REIN: Réseau Epidémiologique et Information en Néphrologie, UKRR: United Kingdom Renal Registry, BMI: body mass index  a: hypertensive nephropathy; b: 2016-2017 | | | | | | |
|  |  |  |  |  |  |  |

**Figure S1.** Percentage of patients transferred from facility based dialysis to peritoneal dialysis (A) and home haemodialysis (B) by month, excluding patients starting on home dialysis, within the first year of haemodialysis initiation.

**(A)**

**(B)**

Results are expressed in percentages, censored for death/transplantation/loss to follow-up/kidney recovery

CORR: Canadian Organ Replacement Registry; REIN: Reseau Epidémiologique et Informatique en Néphrologie ; UKRR : United Kingdom Renal Registry
